# Prevalence of antimalarial drug resistance markers and factors associated with *Plasmodium falciparum* infection in asymptomatic children prior to rectal artesunate implementation in Kapolowe Health District, Democratic Republic of the Congo

**DOI:** 10.1186/s12936-026-06016-6

**Published:** 2026-06-29

**Authors:** Flory Luzolo Khote, Madalena Raposo, Tafadzwa Maseko, Deborah Kanyamukenge, Sebastian Hachizovu, Geofrey Makenga, Eric Mukomena, Aimé Kakudji, Augustin Mutombo, Ghislain Kikunda, Jérémie Sombola, Vivi Maketa, Michael Nambozi, Stephan Duparc, Hans Rietveld, Michael Alifrangis, Helle Hansson, Louise Wellmann, Nathalie Ouare, Christine Manyando, JP Van Geertruyden, Vito Baraka, Hypolite Muhindo Mavoko

**Affiliations:** 1 Department of Tropical Medicine, University of Kinshasa, Kinshasa, Democratic Republic of the Congo; 2 Global Health Institute, University of Antwerp, Antwerp, Belgium; 3 Center for Translational Medicine and Parasitology, Department of Immunology and Microbiology, University of Copenhagen, Copenhagen, Denmark; 4 Department of Infectious Diseases, Copenhagen University Hospital, Copenhagen, Denmark; 5 University of Lubumbashi, Lubumbashi, Democratic Republic of the Congo; 6 National Health Research and Training Institute, Ndola, Zambia; 7 Tanga Centre, National Institute for Medical Research, Hospital Street, P O Box 5004, Tanga, United Republic of Tanzania; 8 MMV Medicines for Malaria Venture, Geneva, Switzerland; 9 Clinical Research Unit of Nanoro, Nanoro, Burkina Faso

**Keywords:** Malaria, *Plasmodium falciparum*, Rectal artesunate, Artemisinin-based combination therapy, Drug resistance, Molecular markers, *Pfkelch13*, Democratic Republic of the Congo

## Abstract

**Background:**

In remote areas where access to parenteral treatment for severe malaria or referral is limited, rectal artesunate (RAS) followed by a full course of artemisinin-based combination therapy (ACT) provided by community health workers would be a pragmatic solution. However, concerns remain that its expanded use may select for artemisinin-resistant *Plasmodium falciparum* strains. This baseline study assessed the presence of molecular markers of resistance to artemisinin and partner drugs and investigated factors associated with *Plasmodium falciparum* infection prior to RAS implementation.

**Methods:**

A community-based cross-sectional survey was conducted in remote areas of the Kapolowe health district, Democratic Republic of the Congo, in March–April 2024. Dried blood spot samples were collected from asymptomatic children aged 6–59 months. Molecular markers of antimalarial drug resistance in *P. falciparum* were analyzed using targeted next-generation sequencing, focusing on the *Pfkelch13, Pfcoronin, Pfubp1, Pfmdr1, and Pfcrt* genes.

**Results:**

Among 1242 children screened in 906 households, 656 (53%, 95% CI: 50–55%) were *P. falciparum* malaria rapid diagnostic test-positive, of which 646 samples were available for analyses. Factors associated with reduced odds of asymptomatic *P. falciparum* infection included higher household socio-economic status and non-agricultural sources of income, whereas children from households where malaria was perceived as treatable had increased odds of infection. No validated *Pfkelch13* mutations associated with artemisinin partial resistance were detected. Several non-synonymous *Pfkelch13* mutations were observed more than once, including A578S (7/395, 1.8%). Two *Pfubp1* mutations associated with reduced dihydroartemisinin susceptibility, D1525E (88/354, 24.9%) and E1528D (62/354, 17.5%) were observed. No resistance-associated *Pfcoronin* mutations were detected. The wild-type *Pfcrt* CVMNK haplotype predominated, while the *Pfmdr1* NFSND haplotype (57/379, 15.0%) associated with reduced lumefantrine susceptibility was identified.

**Conclusions:**

The absence of *Pfkelch13* mutations and a full reversal to wild-type *Pfcrt* are reassuring for directly guiding the implementation strategies of RAS and continued effectiveness of current ACTs. However, the detection of the *Pfmdr1* single mutant N**F**SND haplotype highlights the need for continued monitoring of lumefantrine susceptibility.

**Supplementary Information:**

The online version contains supplementary material available at https://doi.org/10.1186/s12936-026-06016-6.

## Introduction

Malaria, a mosquito-borne infectious disease caused by *Plasmodium* parasites, remains a significant public health challenge in sub-Saharan Africa (SSA) [1]. The World Health Organization (WHO) reports that in 2024, 94% of the world’s 282 million malaria cases and 95% of the world’s 610,000 malaria deaths occurred in the WHO-African region, with children under 5 years of age being the most severely affected [1]. The Democratic Republic of the Congo (DRC) has the second-highest burden of malaria, accounting for 13.3% of global cases and 11.7% of deaths [1, 2]. Important contributors to this are the high transmission intensity, the lack of consistent diagnosis and often difficult access to effective treatment, especially in remote areas [3]. In addition, the displaced populations resulting from the political complexities of the DRC face additional disruptions in healthcare access.

WHO recommends severe malaria to be treated at the hospital with parenteral artesunate followed by a full course of artemisinin-based combination therapy (ACT) for 3 days once it becomes possible to deliver medicines by the oral route [4]. In remote areas where injectable treatment is unavailable, WHO recommends administration of a single pre-referral dose of rectal artesunate (RAS), and thereafter immediate referral of the patient to a facility where intravenous artesunate and other supportive treatments are available [4]. However, it is often difficult for the child to be transported to hospital, given physical distance, impassable roads in the rainy season, lack of transportation, and financial constraints [5]. In this context, the SEMA ReACT project (“Severe Malaria Treatment with RAS and ACT in Remote Settings”) was implemented in remote areas of the DRC and Zambia to evaluate an alternative strategy of integrated Community Case Management (iCCM). Here, when referral to a hospital is not possible, suspected severe malaria is treated by at least one dose of pre-referral RAS followed by a full course of ACT delivered by a community health worker [6]. Given the threat due to artemisinin partial resistance, there is a concern that lack of adherence to the full course of ACT may cause emergence and spread of artemisinin-resistant mutations. This concern is stronger if more than one pre-referral RAS dose is given [1, 7].

Although ACTs remain largely clinically efficacious across SSA, as measured by adequate clinical and parasitological response at day 28, reduced efficacy of artemether–lumefantrine has been reported in some settings, including a recent report from Uganda [8]. Clinical failures in Africa have been associated with resistance markers to ACT partner drugs such as amodiaquine and, more recently, lumefantrine [1, 8]. In addition, artemisinin partial resistance associated with *Plasmodium falciparum* (*P. falciparum*) kelch13 (*Pfkelch13*) mutations causes a significantly increased parasite clearance time which may then result in parasites persisting 72 h after the start of treatment [2, 9, 10]. Artemisinin partial resistance is mediated by ring stage quiescence, a reversible dormancy phenotype when parasites are exposed to artemisinin, hence allowing them to survive and replicate once the drug pressure is removed [11, 12].

Single-nucleotide polymorphisms (SNPs) in the propeller domain of the *Pfkelch13* gene have been associated with delayed parasite clearance and artemisinin partial resistance. These are classified into confirmed mutations, supported by clinical evidence, and candidate mutations, for which clinical data are not yet available. To date, a large number of *Pfkelch13* variants have been validated including F446I, N458Y, C469Y, M476I, Y493H, G533S, R539T, I543T, P553L, R561H, P574L, C580Y, R622I and A675V. Candidate mutations currently include E252Q, P441L, G449A, C469F, A481V, R515K, P527H, N537I/D, G538V, V568G [13, 14]. Furthermore, although the *Pfkelch13* gene is the most widely recognized marker and a key determinant of artemisinin partial resistance, mutations in other genes have been identified as potential artemisinin resistance markers [15]. Notably, mutations in the *P. falciparum* coronin gene *(Pfcoronin)* have been associated with a possible delay in parasite clearance by dihydroartemisinin (DHA). This has been linked to structural similarities between the six-bladed β-propeller domain of *Pfkelch13* and the WD-40 propeller domain of *Pfcoronin* [16–18]. Several mutations have also been identified in the *P. falciparum ubiquitin-specific protease 1* gene (*Pfubp1)*, which have been shown to reduce DHA sensitivity in vitro [19, 20]*.* However, in vivo and field-based evidence linking these genes to clinical outcomes remains limited. Other genes have also been implicated in artemisinin partial resistance, including pfap2-mu [16, 20], and recently identified variants in the PX1 gene have been reported to alter parasite responses to both artemisinin derivatives and lumefantrine [21, 22], although these remain candidate markers requiring further validation.

Mutant alleles of the *P. falciparum* multidrug resistance gene-1 (*Pfmdr1*) and the *P. falciparum* chloroquine resistance transporter gene (*Pfcrt*) convey resistance towards the ACT partner drugs lumefantrine, as well as amodiaquine and piperaquine, respectively [23–26]. Amino acid substitutions in the *Pfmdr-1* gene include N86Y, Y184F, S1034C, N1042D and D1246Y, from which the most frequently reported are N86Y, Y184F and D1246Y [13, 27]. The *Pfcrt* gene harbors SNPs in codons 72–76 of the CVMNK wildtype haplotype. The K76T mutation has been reported to be significantly associated with amodiaquine resistance [13, 28, 29].

The confirmed occurrence of artemisinin partial resistance in the DRC was implicated by the presence of the validated *Pfkelch13* R561H mutation, which is linked to delayed parasite clearance, and the presence of the candidate P441L mutation [2]. The R561H mutation was identified only once in Kabondo (Northern Tshopo Province), while P441L was observed also once in Rutshuru (North Kivu province) [2]. The identification of these mutations in the DRC, together with the high interest in using more than a single dose of RAS monotherapy in remote areas of the Kapolowe health district (KHD), as well as the concerns that monotherapy would lead to an increase in the abundance of artemisinin partial resistance mutations, confirms that there is a need for baseline data and continuous molecular monitoring of markers associated with antimalarial drug resistance. Such monitoring should include both artemisinin resistance markers but also markers for partner drug resistance, since it is generally the partner drug resistance which is linked to clinical failure [30, 31].

To address this urgent monitoring need, a cross-sectional study aimed to determine the baseline prevalence of molecular markers of artemisinin partial resistance (*Pfkelch13*, *Pfcoronin* and *Pfubp1*) as well as those associated with partner drug susceptibility profile (*Pfmdr1* and *Pfcrt*) was conducted in a sentinel population among asymptomatic children under 5 years of age in remote areas of the KHD, before RAS plus ACT intervention. In addition, we investigated factors associated with *P. falciparum* infection in the study population.

## Methods

### Study site and participants

The cross-sectional study was conducted in the KHD of the Haut-Katanga province, in the DRC. This tropical zone bordering Zambia is characterized by perennial malaria transmission and high infection prevalence, in one study measured as 42.7% among children by malaria rapid diagnostic test (mRDT) [32]. The KHD is one of the National Malaria Control Program’s sentinel surveillance sites, and experiences one of the DRC’s highest malaria burdens [32]. The local population is primarily engaged in agriculture, fishing, and artisanal mining. The health district comprises 181 villages (most of them being remote and hard-to-reach) distributed across 16 health areas.

Children aged 6–59 months were actively recruited from randomly selected households during a community survey conducted in March and April of 2024. Blood samples were collected from children whose parents or guardians provided informed consent.

### Data collection procedures

**Fig. 1:**
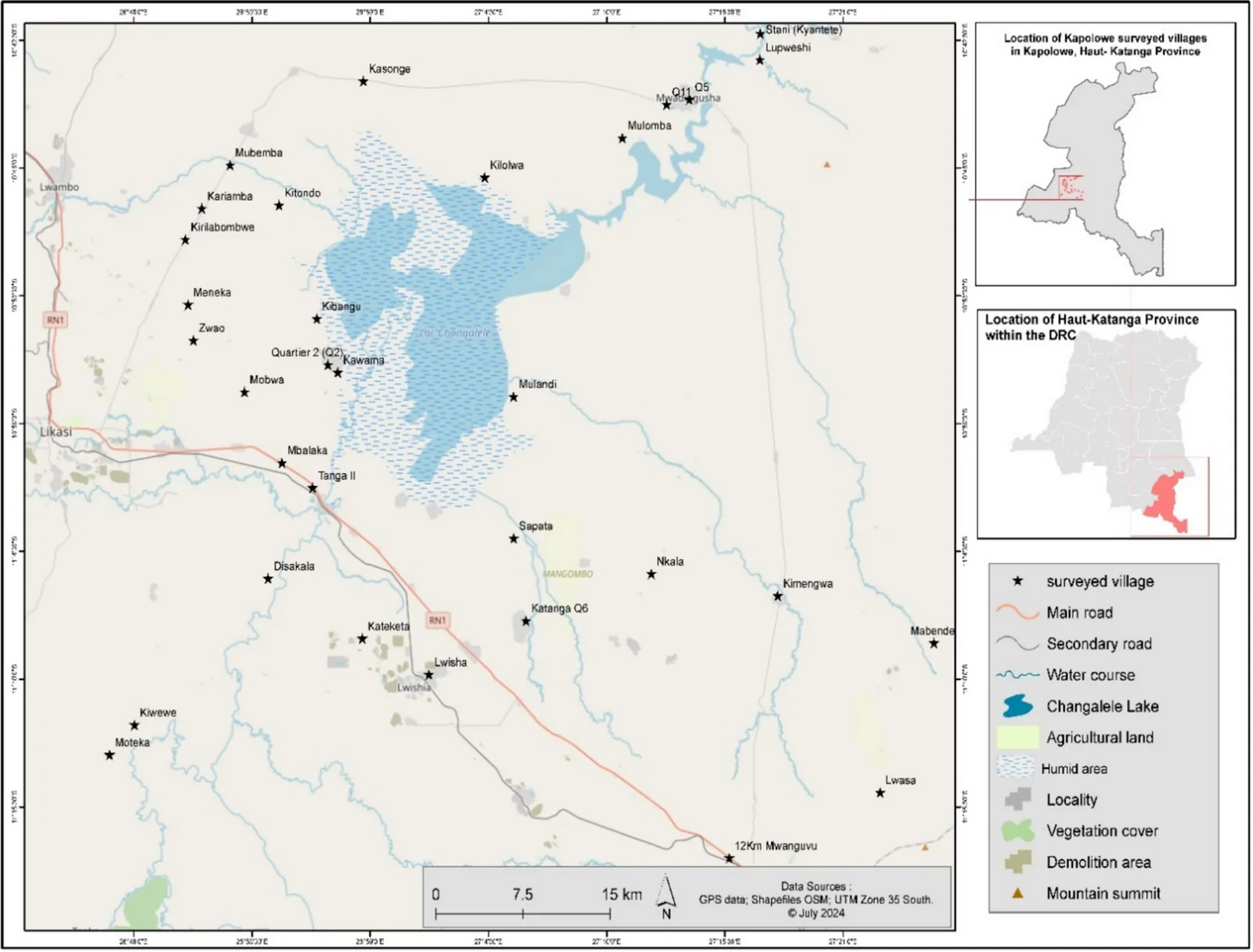
Map of surveyed villages in Kapolowe health district, showing the location of 33 villages grouped into 30 sampling units

This cross-sectional community study was conducted as part of the SEMA ReACT project, which also includes genomic surveillance of *P. falciparum* resistance markers to antimalarial drugs. A two-stage proportional cluster sampling design was employed to obtain a geographically representative sample across the KHD [33]. A total of 30 villages were randomly selected, and within each village, households were selected using the systematic sampling approach. This design yielded a total of 906 consenting households (Fig. 1).

In the eligible households, an informed consent form was provided. After receiving consent from a parent or guardian, capillary blood drops were collected from eligible children for malaria screening using mRDT (*Adv Dx*^*™*^* Paludisme Pf; Lot: ADF124/1222; Mfg. 022–12; Exp: 2024–11*) and additional blood drops were collected and dried on Whatman 3MM filter paper [34], with 3 spots per card. General information on the head of household and eligible children was collected from the household respondent using the case report form collection tool. If a parent or guardian did not provide consent, the household was replaced by the next eligible household in the same village or, if necessary, in a neighbouring village. From 906 eligible households, 1242 asymptomatic children were screened using mRDT, and dried blood spots (DBS) samples were collected. Among them 656 were *P. falciparum* mRDT positive. Each DBS was identified and placed individually in a ziplock bag with silica gel. All children with a positive mRDT result were treated with artesunate-amodiaquine tablets.

### Molecular analysis

#### DNA extraction

All mRDT-positive *P. falciparum* DBS were shipped to the National Institute for Medical Research laboratory in Tanga, Tanzania, where *P. falciparum* DNA was extracted using the commercial Qiagen DNA mini kit extraction [34], according to the manufacturer's instructions. Briefly, one 3-mm DBS punch (approximately 3–5 µL of whole blood) was used for extraction, and DNA was eluted in 50 µL of elution buffer. The eluted DNA was stored at − 80 °C and shipped to the Center for Translational Medicine and Parasitology at the University of Copenhagen (UCPH), Denmark for further analysis.

### Quantitative polymerase chain reaction (qPCR) methodology

At UCPH, a TaqMan-based qPCR assay was performed on each extracted DNA sample to quantify parasite density and confirm mRDT positivity. Reactions were run on an AriaMX qPCR platform using a total volume of 12.5 µL, including 2.5 µL of sample DNA and 10 µL of Master Mix (2.75 µL of H_2_O, 1 µL of Primer/probe mix (final concentration 800 nM/400 nM) and 6.25 µL of Polymerase (Applied Biosystems, Thermo Fisher Scientific, USA)). Primers and probe targeting the multi-copy varATS sequence were obtained from Eurofins Genomics (MWG-Biotech AG, Edersberg, Germany), according to the protocol [35], to enable sensitive detection of *P. falciparum* infection. The qPCR program consisted of the following steps: pre-incubation for 2 min at 50 °C, initial denaturation for 10 min at 95 °C, followed by 45 cycles of denaturation for 15 s at 95 °C and annealing as well as elongation for 1 min at 55 °C (Table 1). All samples with CT value < 40 were considered positive and retained for downstream sequencing analysis.

**Table 1:** TaqMan based qPCR conditions

Primers	PCR conditions	Reagents
Forward (FW) primer: CCC ATA CAC AAC CAA YTG GA	50 °C 2 min	Primer/probe mix:
Reverse (RV) Primer: TTC GCA CAT ATC TCT ATG TCT ATC T	95 °C 10 min	100 µl FW primer + 100 µl RV primer + 50 µl probe + 750 µl H_2_O
Probe: [FAM] TRT TCC ATA AAT GGT [MGBEQ]	(95 °C 15 s, 55 °C 1 min) × 45 cycles	Master Mix: 2.75 µl H_2_O + 1 µl Primer/probe mix (800 nM/400 nM) + 6.25 µl Polymerase

### Amplification of Pfkelch13, Pfmdr1 and Pfcrt genes

**Fig. 2:**
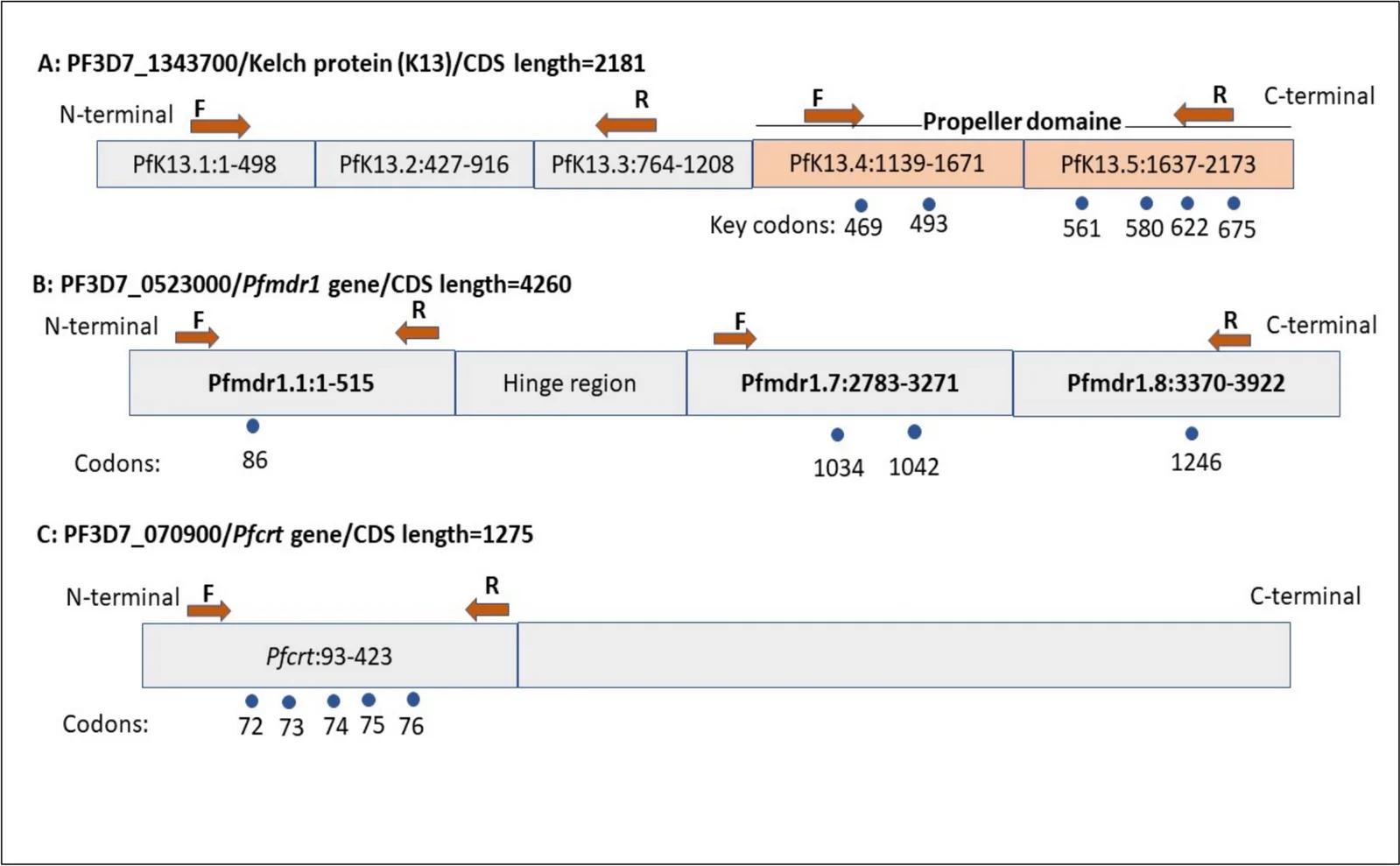
Schematic representation of amplified and sequenced regions in *Pfkelch13*, *Pfmdr1* and *Pfcrt* genes. Legend: A: *Pfkelch13* gene showing the propeller domain with the key codons and primers locations [36]; B: *Pfmdr1* gene showing the amplified fragments with the key codons associated with antimalarial drug resistance and primer locations [36]; C: *Pfcrt* gene showing the amplified fragment with the key codons associated with antimalarial drug resistance and primer locations [36]. Orange arrows indicate inner (nested) primers; F and R denote forward and reverse primers, respectively

A series of outer and nested PCRs were carried out to amplify targeted sequences in *Pfkelch13, Pfmdr1 and Pfcrt* genes (Fig. 2) as described in Nag et al., 2017 and 2019 [36, 37]. All PCR reactions were performed in 2720 Thermocyclers (Applied Biosystems, Thermo Fisher Scientific). Outer PCRs were performed using a total volume of 15 µL, including 1µL of sample DNA and 14 µL of AH PCR Master mix from AH Diagnostics (Århus, Denmark), composed of 2.4 µL of Primer mix (0.32uM), 3 µL of AH pol (5x), and 8.6 µL of H_2_O. The cycling program consisted of initial denaturation for 15 min at 95 °C, 40 cycles of 20 s at 95 °C, 2 min at 51 °C, 2 min at 72 °C and a final extension step lasting 10 min at 72 °C [65]. Nested PCRs used a total volume of 20 µL, including 1µL of outer PCR product and 19 µL of the same master Mix (AH PCR Master mix from AH Diagnostics (Århus, Denmark), composed of 1.3µL of Primer mix (0.13 nM), 4 µL of AH pol (5x), and 13.7 µL of H_2_O. The nested cycling program consisted of 15 min of initial denaturation at 95 °C, 30 cycles of 20 s at 95 °C, 2 min at 52 °C, 2 min at 72 °C and 10 min of final extension at 72 °C [65].

## *Amplification of Pfcoronin* and *Pfubp1 genes*

**Fig. 3:**
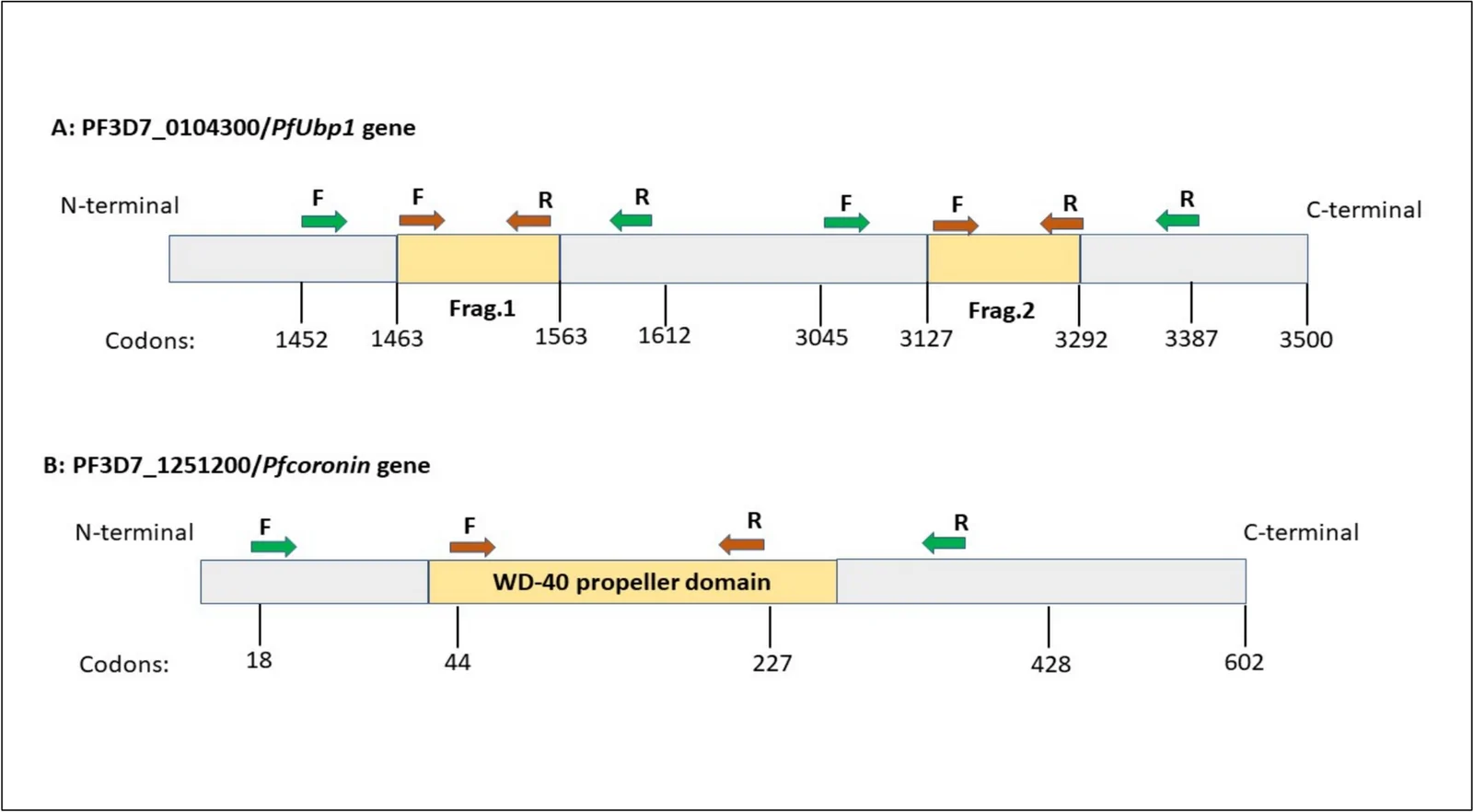
Schematic representation of amplified and sequenced regions in *Pfubp1* and *Pfcoronin* genes. Legend: A: *Pfubp1* (PF3D7_0104300) gene indicating the two amplified fragments (Frag. 1 = fragment 1 and Frag.2 = fragment 2) and primer locations; B: *Pfcoronin* (PF3D7_1251200) gene with the WD40 propeller domain and primer positions. Green arrows indicate outer (primary) primers, and orange arrows indicate inner (nested) primers; F and R denote forward and reverse primers, respectively

Nested PCRs were designed to target specific regions within the *Pfcoronin* and *Pfubp1* genes (Fig. 3) that contain previously described mutations associated with artemisinin resistance [16, 38]. The *Pfcoronin* gene was amplified between codons 44 and 227 (Table 2 and Fig. 3), while the *Pfubp1* gene was amplified in two regions: fragment 1, between codons 1463 and 1563, and fragment 2, between codons 3127 and 3292 (Table 3 and Fig. 3), using 2720 Thermocyclers (Applied Biosystems, Thermo Fisher Scientific). The primers were ordered from Eurofins Genomics (MWG-Biotech AG, Edersberg, Germany) and the AH PCR Master mix from AH Diagnostics (Århus, Denmark).

**Table 2:** PCR conditions for amplification of *Pfcoronin*

Type of PCR	Primers	Product size	PCR conditions	Reagents
Outer PCR	Fw: 5’–CTGTACGGAGACTTGAGGAT – 3’ Rv: 5’ – TGTCTCATGACAGAACCCTT – 3’ Designed by the DRUGS group at UCPH	1423 bp	95 °C 15 min (95 °C 30 s, 56 °C 45 s, 68 °C 30 s) × 35 cycles	1 µl DNA, 2 µl of primer mix (final concentration of Fw and Rv primers: 2 µM),
Nested PCR	Fw: 5’– ATGGCAAGTTGAAGGGGGAG – 3’ Rv: 5’– TTGTCTTCACCACCAAATCCA – 3’ From Ajibaye et al. 2024	542 bp	68 °C 10 min	4 µl AH (5x) polymerase mix, total volume of 20 µl

**Table 3:** PCR conditions for *pfubp1* fragment 1 and fragment 2 amplification

Type of PCR	Primers	Product size	PCR conditions	Reagents
Outer PCR Fragment 1	F: 5’-CGCCCGTACTATGAAGAAGATC-3’ R: 5’-GGCTTTTACCTGAACTGTTCAGG-3’ From Henriques et al. 2014	484 bp	94 °C 15 min (94 °C 30 s, 55 °C 45 s, 68 °C 30 s) × 30 cycles 68 °C 10 min	1 µl DNA, 1 µl primer mix (final concentration of Fw and Rv primers: 2 µM), 4 µl AH (5x) polymerase mix, total volume of 20 µl
Outer PCR Fragment 2	F:5’-CGGCTCAGACCAACATGATC-3’ R: 5’-CGGTTAAGAATTAATATCAAGTGAC-3’ Designed by the DRUGS group at UCPH	1026 bp	94 °C 15 min (94 °C 30 s, 55 °C 45 s, 62 °C 30 s) × 30 cycles 62 °C 10 min	1 µl DNA, 2 µl primer mix (final concentration of Fw and Rv primers: 2 µM), 4 µl AH (5x) polymerase mix, total volume of 20 µl
Nested PCR Fragment 1	F: 5’-CGTAAACAGAATATTCAGGATTGC-3’ R: 5’-CTAGCCCTTTATTATCATTATCG-3’ From Henriques et al. 2014	304 bp	94 °C 15 min (94 °C 30 s, 55 °C 45 s, 68 °C 30 s) × 30 cycles 68 °C 10 min	1 µl DNA, 2 µl primer mix (final concentration of Fw and Rv primers: 2 µM), 4 µl AH (5x) polymerase mix, total volume of 20 µl
Nested PCR Fragment 2	F: 5’-ACCGTGTGAGTCCCAATAAT-3’ R: 5’-TCTTTTTCTGAACCACCTAA-3’ Designed by the DRUGS group at UCPH	496 bp	94 °C 15 min (94 °C 30 s, 57 °C 45 s, 62 °C 30 s) × 30 cycles 62 °C 10 min	1 µl DNA, 2 µl primer mix (final concentration of Fw and Rv primers: 2 µM), 4 µl AH (5x) polymerase mix, total volume of 20 µl

### Agarose gel electrophoresis

Agarose gel electrophoresis was performed to confirm successful amplification of nested PCR products and to assess potential contamination using negative (no-template) controls before considering samples valid for downstream analysis. PCR products were resolved on 1.5% agarose gels prepared in 0.5 × TBE buffer. Electrophoresis was performed under standard conditions (130–210 mV, 100–210 mA, 30–60 min) adjusted according to gel size. Gels were visualized using Ethidium Bromide staining on a ChemiDoc MP Imaging System (Bio-Rad).

### Index PCRs and Miseq run

Next, index PCRs were performed on pools of nested PCR products according to each sample plate. Briefly, 1 µL of pooled PCR product was amplified in a total reaction volume of 11 µL containing 3 µL Illumina-compatible index primers (i5 and i7) and a high-fidelity PCR master mix. The cycling conditions consisted of 15 min of initial denaturation at 95 °C, followed by 20 cycles of 20 s at 95 °C, 1 min at 60 °C, and 2 min at 72 °C [65]. All nested amplicons included 5’ adapter overhang sequences, enabling the addition of unique dual indices during the index PCR. These indices consisted of unique 8-basepair barcode sequences, allowing multiplexing and accurate identification of each sample. Pooling ensured that all fragments amplified from the same sample received the same index combination, enabling traceability of each amplicon to the corresponding patient [36].

Indexed PCR products were purified using 175 µL of Dynabeads magnetic beads (Invitrogen, ThermoFisher Scientific), according to standard protocols, and subsequently normalized to a final concentration of 4 nM. Library quality and concentration were assessed prior to sequencing.

A 5% PhiX control library (Illumina, California, USA) was spiked in to increase nucleotide diversity caused by the high AT content in the *P. falciparum* genome. Sequencing by synthesis was then performed using the Illumina MiSeq platform with the Miseq Reagent V3 Kit (2 × 300 bp) (Illumina, California, USA), according to the manufacturer’s instructions.

Negative controls were included during indexing and library preparation to monitor potential cross-contamination.

### Quality control of Illumina reads

After sequencing, samples were sorted into folders matching their original extracted DNA plates according to index sequences and run through the Galaxy website (http://www.usegalaxy.fr) for quality control and trimming. FastQC and MultiQC reports were generated to evaluate the quality of the data [39, 40]. The Trimmomatic tool was used to trim the reads, removing sequences with Phred scores below 30 (< 99.9% base-calling accuracy) [41]. The trimmed sequences (R1 and R2 files) were then aligned to the *P. falciparum* reference genome 3D7 using a custom Python script, executed in PyCharm [36, 37]. No dedicated variant-calling software (e.g., GATK or FreeBayes) was used. Variants were identified directly from the aligned sequence data using custom R scripts and predefined filtering thresholds [36, 37]. Variants were identified at each position relative to the reference sequence. This data was then imported into Microsoft Excel using an R script developed by Max Benjamin Sauerland and Helle Hansson at the UCPH (The R program is not currently publicly available). The R script set a cut-off at 50 reads, only SNPs with at least 50 × depth in specific position, and ≥ 90–95% coverage of the targeted amplicon sequence were considered for downstream analysis. Mixed infections were identified at positions where two or more nucleotides were each supported by at least 20% of reads [36].

### Statistical analysis

Data were analyzed using Microsoft Excel 2021 (version 2108) and R version 1. A two‑stage cluster design (villages, then households) was used to recruit study participants. Descriptive estimates for sociodemographic characteristics of household respondents and children, and the proportion of *P. falciparum* mRDT-positive were derived using survey‑weighted methods that account for clustering at village level and clustering at both village and household levels. Weighted proportions and their 95% confidence intervals (CIs) are presented alongside unweighted estimates to allow comparison.

To explore factors associated with *P. falciparum* infections, survey-weighted logistic regression models were fitted with mRDT positivity as the binary outcome. Candidate predictors included household respondent sociodemographic characteristics, household socio‑economic status, malaria‑related knowledge and perceptions, and access to healthcare. Univariable models were first run for each covariate, and variables showing evidence of association (p<0.20) or considered *a priori* relevant were included in a multivariable model. Odds ratios (ORs) with 95% CIs and p‑values were obtained from survey‑weighted models, accounting for sampling weights.

For molecular analyses, only mRDT‑positive samples that were qPCR‑confirmed and successfully sequenced were included. Because this group represents a selected subset of infected children rather than a probability sample of all children in KHD, frequencies of individual mutations and haplotypes were calculated as unweighted proportions among sequenced samples, with binomial 95% CIs for each gene. Sequencing success (per gene) was summarized as the proportion of qPCR‑positive samples that met pre‑defined read‑depth and coverage thresholds.

## Results

### Sociodemographic characteristics and *P. falciparum*-positive samples available for analysis (general information)

A total of 1242 DBS samples were collected from asymptomatic children screened in 906 eligible households, including 656 *P. falciparum* mRDT-positive samples (unweighted 53% (50–55%), weighted 51% (44–58%)). The unweighted proportion (53, 95% CI: 50–55%) was retained for molecular analysis. From the 656 *P. falciparum* mRDT-positive DBS samples, 10 were lost, resulting in 646 DBS samples available and retained for this study. Females accounted for 329 of 646 children (50.9% [95% CI: 47.1–54.8%]). The median age of children was 36 months (IQR: 24, 47 months). Most of the 906 household respondents were female (768/906; 85% [95%CI: 83–87%]), with a median age of 29 years (IQR: 30, 32 years). Overall, 20% (95% CI: 15–25%) of household respondents reported no formal education, while 460/906; 50% (95% CI: 45–55%) had completed primary schooling. The main livelihood activities of households were fishing, agriculture and cash crop sales (747/906; 80% [95%CI: 68–88%]) (Table 5).

**Table 5:** Sociodemographic characteristics of household respondents in a cross-sectional survey in Kapolowe health district in 2024

Variable	N	Unweighted proportion (95% CI)^1^	Weighted proportion (95% CI)^1^
Sex	906		
Female		85 (82–87)	85 (83–87)
Male		15 (13–18)	15 (13–17)
Marital status	904		
Married		95 (93–96)	94 (93–96)
Divorced		2.7 (1.6–3.7)	3.0 (2.1–4.3)
Single		1.2 (0.50–1.9)	1.3 (0.83–1.9)
Widowed		1.4 (0.66–2.2)	1.3 (0.69–2.6)
Education level	906		
None		21 (18–23)	20 (15–25)
Primary		51 (48–54)	50 (45–55)
Secondary or higher		28 (26–31)	31 (25–37)
Age	906	29 (24, 37)	29 (24, 37) (30, 32)
Asset-based socio-economic quintiles	906		
Low		33 (30–36)	32 (25–39)
Middle		33 (30–36)	34 (30–39)
High		33 (30–36)	34 (29–40)
Main income of the household	906		
Fishing/Agriculture/Cash Crop Sales		82 (80–85)	80 (68–88)
Commerce/Shop		5.8 (4.3–7.4)	6.6 (3.6–12)
Civil servant		3.3 (2.1–4.5)	3.5 (2.2–5.5)
Other		8.5 (6.7–10)	10 (4.8–20)
Nearest health facility	900		
Private/Other drug sellers		23 (20–25)	24 (16–34)
Public health post		64 (61–67)	65 (51–76)
Public hospital		1.7 (0.83–2.5)	1.8 (0.87–3.7)
Community health worker		11 (9.4–14)	9.6 (4.5–19)
Heard malaria message	902		
No		47 (44–50)	47 (42–52)
Yes		53 (50–56)	53 (48–58)
Perception of malaria	902		
Not a serious health problem		3.8 (2.5–5.0)	3.0 (2.1–4.4)
Somewhat serious		12 (10–15)	14 (11–18)
Very serious		84 (81–86)	83 (79–87)
Is malaria treatable	893		
No		18 (16–21)	19 (15–23)
Yes		82 (79–84)	81 (77–85)

^1^%; Median (Q1, Q3)

*CI* Confidence interval

### Prevalence of infection and associated factors

In the multivariate analysis of full screened children (*N* = 1242), household respondents with secondary or higher education had lower odds of child infection compared with those with no education (adjusted OR: 0.6, 95%CI: 0.4–1.00; *p*≈0.05). Household respondents whose main income source was civil servant or other non-agriculture activities had reduced odds of child infection compared with those engaged in fishing, agriculture, cash crop sales, or commerce (civil servant: adjOR:0.5, 95%CI:0.2–1.2; *p*≈0.1; other: adjOR:0.4, 95% CI 0.2–0.9; *p*≈0.03). Children from middle and high socio‑economic households also had reduced odds of infection compared with those from the lowest quintile (middle: adjOR: 0.7, 95%CI: 0.5–0.9; *p* = 0.04; high: adjOR: 0.6, 95%CI: 0.4–0.9; p = 0.02). Household respondents who believed malaria is treatable had higher odds of having an infected child than those who did not (adjOR: 2.2, 95% CI 1.4–3.5; *p*≈0.004) (Supplementary Table S1).

### Amplicon sequencing success rate and analysis cohort

From 646 mRDT-positive *P. falciparum* DBS samples, 538 (83.3% [95% CI: 80.3–86.1%]) were confirmed positive by qPCR with detectable levels of *P. falciparum* DNA and formed the final cohort for drug resistance markers analysis (data not shown).

### Frequencies of key drug resistance mutations

#### a. Frequency of mutations in the *Pfkelch13* gene

Of the 538 samples sequenced, 395 (73.4% [95% CI: 69.5–76.1]) resulted in complete sequence data covering the targeted *Pfkelch13* gene region. None of the successfully sequenced samples carried validated or candidate mutations associated with artemisinin partial resistance according to the WHO [1]. A complete list of all detected non-synonymous (NS) mutations, including single-occurrence variants, is provided in Supplementary Table S2. Eight recurrent NS mutations observed more than once among the successfully sequenced samples are highlighted below to focus on variants with higher recurrence and potential epidemiological relevance: K189T (78/395, 19.8% [95% CI: 16.1–23.1]); K189N (12/395, 3.0% [95% CI: 1.8–5.2]); L258M (10/395, 2.5% [95% CI: 1.4–4.6]); H136N (8/395, 2.0% [95% CI: 1.0–3.1]); R255K (8/395, 2.0% [95% CI: 1.0–3.1]), A578S (7/395, 1.8% [95% CI: 0.9–3.6]); V83I (2/395, 0.5% [95% CI: 0.1–1.8]) and K124N (2/395, 0.5% [95% CI: 0.1–1.8]) (Table 6).

**Table 6:** Frequencies of non-synonymous and synonymous mutations detected more than once in sequenced samples of *Pfkelch13*, *Pfcoronin*, and *Pfubp1 genes*

Gene	Mutation (non-synonymous)	N (%)	95% CI	Mutation (synonymous)	N (%)	95% CI
*PfKelch13*	K189T	78 (19.8)	16.1–23.1	P417P	8 (2.0)	1.0–3.9
	K189N	12 (3.0)	1.8–5.2	R471R	5 (1.3)	0.5–2.9
	L258M	10 (2.5)	1.4–4.6	L119L	5 (1.3)	0.5–2.9
	H136N	8 (2.0)	1.0–3.1			
	R255K	8 (2.0)	1.0–3.1			
	A578S	7 (1.8)	0.9–3.6			
	V83I	2 (0.5)	0.1–1.8			
	K124N	2 (0.5)	0.1–1.8			
*Pfcoronin*	S183G ^(a)^	397 (93.4)	90.6–95.4	K212K	425 (100)	
	P76S ^(a)^	14 (3.3)	1.1–5.5	S204S	8 (1.9)	0.9–3.7
	V62M ^(a)^	7 (1.7)	0.8–3.4	H208H	4 (0.9)	0.4–2.4
*Pfubp1*	W1470C	170 (48.0)	42.9–53.2	D1534E	108 (30.5)	25.9–35.5
	D1525E ^(b)^	88 (24.9)	20.6–29.6	Y3219Y	21 (5.9)	3.9–8.9
	E1528D ^(b)^	62 (17.5)	13.9–21.8	N3245N	5 (1.4)	0.6–3.2
	E1531D	58 (16.4)	12.9–20.6	N1515N ^(a)^	4 (1.1)	0.4–2.9
	Y1556C	6 (1.7)	0.8–3.7	N1518N	25 (7.1)	4.8–10.2
	S1472Y	4 (1.1)	0.4–2.9	H208H	4 (1.1)	0.4–2.9
	N1518Y	4 (1.1)	0.4–2.9	N3245N	5 (1.4)	0.6–3.3
	E1519D	4 (1.1)	0.4–2.9	N1515N	4 (1.1)	0.4–2.9
	G1513E	3 (0.9)	0.3–2.5			
	D1522E	3 (0.9)	0.3–2.5			
	D1539G	2 (0.6)	0.2–2.0			
	T3212M	2 (0.6)	0.2–2.0			
	H3224Y	2 (0.6)	0.2–2.0			
	Y1521N	2 (0.6)	0.2–2.0			

^(a)^ Previously published mutations not associated with reduced artemisinin susceptibility [17, 42]

^(b)^ Have been associated with reduced DHA susceptibility [20, 43]

*CI* confidence interval

#### b. Frequency of mutations in the *Pfcoronin* gene

Among the 425/538 (79.0% [95% CI: 75.4–82.2%]) successfully sequenced samples for this gene, 397 (93.4% [95% CI: 90.6–95.4%]), 14 (3.3% [95% CI: 1.1–5.5%]), and 7 (1.7% [95% CI: 0.8–3.4%]) carried the NS mutations S183G, P76S, and V62M, respectively (Table 6).

#### c. Frequency of mutations in the *Pfubp1* gene

Out of 354/538 (65.8% [95% CI: 61.7–69.7%]) successfully sequenced samples for *Pfubp1* gene, 170 (48.0% [95% CI: 42.9–53.2%), 88 (24.9% [95% CI: 20.6–29.6%]), 62 (17.5% [95% CI: 13.9–21.8%]) and 58 (16.4% [95% CI: 12.9–20.6%]) carried the W1470C, D1525E, E1528D and E1531D mutations, respectively. Other NS mutations, distinct from W1470C, D1525E, E1528D and E1531D, were also detected more than once in the successfully sequenced samples (Table 6).

#### d. Frequency of haplotype mutations in the *Pfcrt and Pfmdr1* genes

In codon 72–76 of the *Pfcrt* gene only the wild-type CVMNK haplotype was detected in all sequenced samples (100%).

For the *Pfmdr1* gene, the codon 86–184-1034-1042-1246 were sequenced. Wild-type NYSND haplotype predominated in 206/379 (54.4% [95%CI: 49.3–59.3%]) successfully sequenced samples. Among the mutant haplotypes detected, the NFSND haplotype driven by the 184 F mutant genotype was the most frequently observed in 57/379 (15.0% [95%CI: 11.8–18.1%]) successfully sequenced samples. Mixed genotypes involving codons 86, 184, and 1246 were also detected (Table 7).

**Table 7:** Allele and haplotype frequencies of the *pfmdr1* gene (N = 379)

*Pfmdr1* position	Allele	n	Frequency (%)	95% CI
86	N	370	97.6	95.6–98.8
	Y	2	0.5	0.1–1.9
	NY*	7	1.9	0.9–3.8
184	F	57	15.0	11.8–18.1
	Y	213	56.2	51.2–61.1
	FY*	109	28.8	24.4–33.5
1034	S	379	100	–
1042	N	379	100	–
1246	D	375	98.9	97.3–99.6
	Y	2	0.5	0.1–1.9
	YD*	2	0.5	0.1–1.9
*Pfmdr1* haplotype	–	n	Frequency (%)	95% CI
NYSND (WT)	–	206	54.4	49.3–59.3
NFSND (1)	–	57	15.0	11.8–18.1
NYSNY (1)	–	1	0.3	0.1–1.5
YYSND (1)	–	1	0.3	0.1–1.5
YFSNY (3)	–	1	0.3	0.1–1.5

^*^Mixed genotypes

*Pfmdr1* haplotypes frequencies (codons 86–184–1034–1042-1246): Mutants in bold: NYSND, NFSND, NYSNY, YFSNY, YYSND. The number next to the haplotypes indicate the amount of mutations, while WT is indicative of wild-type haplotype.

## Discussion

This study provides a baseline assessment of the molecular markers of antimalarial drug resistance in the KHD, an area targeted for the planned implementation of RAS plus ACTs. This assessment is not exhaustive, as some recently proposed candidate markers, including PX1 gene associated with decreased susceptibility to both artemisinin derivatives and lumefantrine, were not included [21, 22]. The absence of WHO-validated *Pfkelch13* mutations suggests no evidence of *Pfkelch13*-mediated artemisinin resistance. However, the fixation of the wild-type *Pfcrt* haplotype should be interpreted with caution, as its impact on drug susceptibility may vary depending on the partner drug. Notably, wild-type *Pfcrt*, often in combination with *pfmdr1* variants, has been associated with reduced susceptibility to lumefantrine [44]. In addition, the presence of the *Pfmdr1* mutant haplotype NFSND, which has been associated with altered susceptibility to lumefantrine, further highlights the need for continued molecular surveillance. Overall, these findings suggest that the *P. falciparum* population remains largely susceptible to artemisinin derivatives.

In light of the emergence and spread *of P. falciparum* ACTs drug resistance [1, 2, 45–47], there is concern that scaling up of repeated doses of RAS without completing the full ACT course in remote areas may increase drug pressure and promote the selection of resistant strains to artemisinin derivatives [7]. Thus, the WHO recommendation to strengthen ongoing molecular surveillance is essential to monitor the distribution of antimalarial drug resistance markers [1].

Current surveillance relies largely on therapeutic efficacy studies in clinical settings, overlooking the hidden reservoir of asymptomatic carriers in hyperendemic areas. These asymptomatic carriers may sustain long-term infections and continuously transmit resistant clones [48, 49]. However, while surveillance of asymptomatic carriers is valuable for investigating drug resistance within this hidden malaria reservoir, it should be acknowledged that ACTs are primarily used to treat uncomplicated symptomatic malaria, whereas RAS is reserved as a pre-referral treatment for severe cases. As such, asymptomatic infections are typically not treated in routine clinical practice, although they may still contribute to the persistence and spread of resistant parasites. In addition, parasite genetics remain a key factor in *P. falciparum* resistance, particularly in remote areas where limited access to healthcare increases vulnerability and facilitates the spread of resistant strains [1, 50].

The *Pfkelch13* findings are operationally reassuring, as it suggests the artemisinin and derivatives component of both RAS and the accompanying ACT should retain high efficacy, reducing the risk of early treatment failure during delivery of RAS for community-based management of severe malaria.

Among the six NS mutations identified in the *Pfkelch13* gene, none corresponded to the validated or candidate mutations associated with artemisinin partial resistance according to WHO [1, 51], consistent with previous findings reported by Mesia et al. in the same KHD [2]. Similar findings have also been reported by Sitali et al. in Zambia, a country directly neighboring the KHD [52]. The latter of relevance as the selection of resistant mutations between neighboring countries depends, among other factors, on treatment policies and cross-border population flows [53]. This suggests that, despite sporadic reports of mutations linked to artemisinin partial resistance [2, 54], their distribution remains limited and ACTs, as well as RAS monotherapy, are likely still effective in treating children with severe malaria in remote areas. The A578S mutation was the only NS mutation located within the propeller region of *Pfkelch13* gene observed in our samples, consistent with reports from other areas of the country [55] and being one of the most frequently reported in Africa [52, 56]. Although not associated with artemisinin partial resistance clinically or in vitro, its presence, even at low frequency, and its proximity to C580Y highlight the need for continued monitoring [55].

While the three mutations, G50E, R100K, and E107V previously reported to be associated with reduced DHA susceptibility in vitro [42] were not observed in *Pfcoronin* gene, we identified the S183G, P76S, and V62M mutations, which have been described in African isolates, but have yet to be associated with reduced artemisinin susceptibility [17, 42]. By contrast, the *Pfubp1* D1525E and E1528D mutations previously reported to be associated with reduced DHA susceptibility in clinical isolates [20, 43, 57], were observed in our study. While the *Pfubp1* mutations are frequently reported in Africa [38], their clinical significance and role as compensatory or independent driver of artemisinin partial resistance remains unknown. As *Pfubp1* has functional similarity to *Pfkelch13* in hemoglobin endocytosis [19], the detection of these mutations in our study underlines the need to intensify the surveillance of both *Pfubp1* as well as *Pfcoronin* to track their evolution alongside *Pfkelch13*. Furthermore, implementation of genotype–phenotype association studies to clearly determine the marker’s association with delayed parasite clearance in vivo*,* and to raise the possibility to include them as novel candidate markers beyond *Pfkelch13* in future surveillance strategies is needed [20, 38, 43].

The haplotype frequencies based on codons 72–76 of the *Pfcrt* gene in this study consisted exclusively of the wild-type CVMNK haplotype, which is similar to the findings reported by Mesia et al. in the same area [2]. This profile reflects the absence of mutant haplotypes associated with reduced chloroquine (CQ) susceptibility following the DRC’s withdrawal from previous malaria treatment guidelines, given that the main mutant CVIET haplotype is no longer circulating. This observation further confirms the previously observed reversion to CQ sensitivity, suggesting susceptibility to amodiaquine in the study area [2]. Similar reports have indicated recovery of CQ-susceptibility in Malawi, Tanzania, Kenya and Mozambique, following its withdrawal from previous treatment guidelines [58]. This trend may also reflect selection for parasites carrying the wild-type CVMNK haplotype due to the widespread use of lumefantrine in the artemether-lumefantrine (AL) combination [24]. These findings are highly encouraging for malaria control, suggesting an indirect benefit for the sustained efficacy of ACTs, as it reduces the likelihood of cross-resistance involving *Pfcrt* and *Pfmdr1* genes [24]. The complete reversal of mutant *Pfcrt* haplotypes to wild-type suggests that amodiaquine (deployed as artesunate-amodiaquine combination), the drug recommended in the many African countries’ treatment guidelines, should retain full efficacy, providing a therapeutic option. Moreover, it highlights the success of treatment policy changes, demonstrating that removing drug pressure can reverse the trend of resistant parasite populations [1, 59, 60].

In this study, the analysis of the *Pfmdr1* gene revealed that the wild-type NYSND haplotype predominated in the sequenced samples, suggesting that treatments with various ACTs remain largely effective in the study area. Among the mutant haplotypes found, the N**F**SND haplotype was the most frequently reported. This observation is consistent with a previous study in the same area [2]. This *Pfmdr1* N**F**SND haplotype is associated with reduced lumefantrine susceptibility, and is detected at a relatively high frequency in almost the entire country [2], as well as in several other African countries where lumefantrine has been introduced as first-line partner drug in the AL combination [23, 27]. AL is the most widely available ACT in private pharmacies, where locals resorts to self-medication (otherwise, non-adherence to national guidelines), thereby increasing the drug pressure on parasites [1, 61]. Although the overall distribution observed is dominated by the wild-type haplotype, the occurrence of the *Pfmdr1* NFSND mutant haplotype highlights the need for continued molecular surveillance. Potential changes in treatment policy and private sector pharmaceutical regulations on drug use could anticipate and mitigate potential changes in drug resistance towards ACTs [1]. The new strategy of multiple first line treatments (MFT), could be a solution to reduce drug pressure and risk of selective of *P. falciparum* resistant strains [60]. Beyond MFT, promising options include the development of triple artemisinin-based combination therapies and recently novel non-artemisinin derivative agents such as ganaplacide/lumefantrine combination (Novartis). By integrating these options in future treatment policies, it could diversify antimalarial pressure and prolong the life of current ACTs [62, 63].

Beyond molecular findings, socio-economic determinants were significantly associated with child infection risk and may indirectly influence antimalarial drug resistance dynamics [64]. Higher household respondent education, improved socio-economic status, and non-agricultural livelihoods were associated with reduced odds of child infection, potentially limiting drug exposure and selection pressure on circulating parasites. Conversely, household respondents who perceived malaria as readily treatable showed higher odds of child infection. This may reflect a lower perceived severity of the disease, leading to reduced use of preventive measures and increased reliance on self-medication or delayed care-seeking. Such practices, often occurring outside formal health systems, may result in incomplete or inappropriate treatment, allowing parasites to persist in infected individuals. This persistent parasitemia can contribute to sustained transmission within the community, thereby increasing the risk of infection in children [64]. Such patterns may contribute to heterogeneous drug pressure and help explain the persistence of certain partner-drug resistance haplotypes despite the absence of WHO-validated *Pfkelch13* mutations in our study. These findings underscore the importance of integrating socio-behavioral factors into molecular surveillance frameworks.

## Strengths and weaknesses

This study provides valuable baseline molecular data on antimalarial resistance markers among asymptomatic children in remote areas prior to RAS implementation. However, its cross-sectional design, its geographic scope limited to a single district, and the lack of direct clinical correlates warrant cautious interpretation and highlight the need for longitudinal post-intervention surveillance. Moreover, the focus on a limited set of molecular markers does not capture the full complexity of resistance, as additional known and yet unidentified genetic mechanisms may also contribute to reduced susceptibility to artemisinin derivatives and partner drugs.

## Conclusion

This molecular surveillance study confirmed the absence of WHO-validated *Pfkelch13* mutations, indicating continued susceptibility to artemisinin-based drugs. Complete reversion to the CQ sensitive *Pfcrt CVMNK* haplotype observed suggests susceptibility to amodiaquine. Furthermore, the detection of the *Pfmdr1* single mutant N**F**SND haplotype highlights the need to maintain continuous monitoring of lumefantrine susceptibility in the area, as AL is the most widely used ACT in the health district. Overall, the findings suggest a favorable resistance landscape for the continued use of ACTs and implementation of RAS in the KHD. Therefore, sustained molecular surveillance of antimalarial resistance markers is critical for the RAS-plus-ACT strategy to safeguard its long-term effectiveness.

## Supplementary Information

Supplementary Material 1

Supplementary Material 2

Supplementary Material 3

## Data Availability

The sequencing data generated in this study have been registered in the European Nucleotide Archive (ENA) under accession number PRJEB111777. Sample accession numbers (ERS) are provided in Supplementary Table S3. The raw sequencing reads (ERR) have been submitted to ENA and will be released upon publication of the manuscript, in accordance with ENA data release policies.

## List of abbreviations

ACT: Artemisinin-based combination therapy
adjOR: Adjusted odds ratio
AL: Artemether–lumefantrine
ENA: European Nucleotide Archive
ERS: European read sample
CI: Confidence interval
CT: Cycle Threshold
DBS: Dried blood spots
DHA: Dihydroartemisinin
DNA: Deoxyribonucleic acid
DRC: Democratic Republic of the Congo
FW: Forward primer
iCCM: Integrated Community Case Management
KHD: Kapolowe health district
mRDT: Malaria rapid diagnostic test
MFT: Multiple First-Line Treatments
N: Number
NS: Non-synonymous
OR: Odds Ratio
P. falciparum: Plasmodium falciparum
*Pfcoronin*: *Plasmodium falciparum* coronin gene
*Pfcrt*: *Plasmodium falciparum* chloroquine resistance transporter gene
*Pfkelch13*: *Plasmodium falciparum* kelch 13 gene
*Pfmdr1*: *Plasmodium falciparum* multidrug resistance 1 gene
qPCR: Quantitative Polymerase Chain Reaction
RAS: Rectal artesunate
RV: Reverse primer
SEMA ReACT: Severe malaria treatment with rectal artesunate plus artemisinin-based combination therapy
SNPs: Single Nucleotide Polymorphisms
SSA: Sub-Saharan Africa
WHO: World Health Organization
