# Supplementary material for: Prevalence of antimalarial drug resistance markers and factors associated with Plasmodium falciparum infection in asymptomatic children prior to rectal artesunate implementation in Kapolowe Health District, Democratic Republic of the Congo
Source: Malar J. 2026 Jun 29;25:332. doi: 10.1186/s12936-026-06016-6 (PMC13584420; doi:10.1186/s12936-026-06016-6)
Supplement: Supplementary file 1 [file 12936_2026_6016_MOESM1_ESM.docx]

Table S1. Multivariate analysis of household socio-demographic characteristics associated with P. falciparum infection detected by mRDT among children

| variable | N | Unweighted mRDT positive (95% CI)^1^ | Weighted mRDT positive (95% CI)^1^ | Crude OR (95% ci) | p-value | Adjusted OR (95% ci) | p-value |
| --- | --- | --- | --- | --- | --- | --- | --- |
| **Sex of household respondent** | 1,242 |  |  |  |  |  |  |
| Female |  | 54 (51–57) | 52 (44 – 60) | 1 |  | 1 |  |
| Male |  | 48 (41–55) | 46 (37 – 54) | 0.8 (0.6-1.1) | 0.2 |  |  |
| **Marital status of household respondent** | 1,239 |  |  |  |  |  |  |
| Married |  | 53 (50–56) | 51 (45 – 58) | 1 |  | 1 |  |
| Not married |  | 43 (31–56) | 41 (24 – 58) | 0.7 (0.4-1.2) | 0.2 |  |  |
| **Education level of household respondent** | 1,242 |  |  |  |  |  |  |
| No education |  | 56 (50–62) | 56 (48 – 64) | 1 |  | 1 |  |
| Primary education |  | 61 (57–64) | 58 (52 – 64) | 1.1 (0.7-1.6) | 0.7 | 1.2 (0.8-1.9) | 0.3 |
| Secondary or higher |  | 38 (33–43) | 37 (26 – 48) | 0.5 (0.3-0.8) | **0.01** | **0.6 (0.4-1.0)** | **0.05** |
| **Age of household respondent** | 1,242 | 30 (24, 38) | 30 (24, 38) | 1.0 (1.0-1.03) | 0.1 | 1.0 (0.1-1.0) | 0.1 |
| **Number of people in the household** | 1,231 | 6 (5, 8) | 6(4, 8) | 0.1 (0.9-1.1) | 0.5 |  |  |
| **Asset-based socio-economic quintiles** | 1,242 |  |  |  |  |  |  |
| Low |  | 63 (58–68) | 62 (57 – 67) | 1 |  | 1 |  |
| Middle |  | 50 (46–55) | 49 (39 – 59) | 0.6 (0.4-0.9) | **0.02** | 0.7(0.5-0.9) | **0.04** |
| High |  | 46 (42–51) | 43 (33 – 54) | 0.5 (0.3-0.7) | **0.003** | 0.6 (0.4-0.9) | **0.02** |
| **Main income of the household** | 1,242 |  |  |  |  |  |  |
| Fishing/Agriculture/Cash Crop Sales |  | 57 (54–60) | 55 (50 – 60) | 1 |  | 1 |  |
| Commerce/Shop |  | 43 (31–54) | 42 (23 – 61) | 0.6 (0.3-1.3) | 0.16 | 0.6 (0.3-1.3) | 0.2 |
| Civil servant |  | 33 (19–48) | 32 (13 – 50) | 0.4 (0.2-0.9) | **0.04** | 0.5 (0.2-1.2) | 0.1 |
| Other |  | 31 (22–39) | 28 (10 – 47) | 0.3 (0.1-0.8) | **0.01** | 0.4 (0.2-0.9) | **0.03** |
| **Nearest health facility** | 1,231 |  |  |  |  |  |  |
| Private/Other drug sellers |  | 46 (40–51) | 45 (28 – 63) | 1 |  |  |  |
| Public health post |  | 56 (53–60) | 53 (48 – 59) | 1.4 (0.7-2.7) | 0.3 |  |  |
| Public hospital |  | 52 (32–73) | 57 (20 – 93) | 1.6 (0.3-9.9) | 0.6 |  |  |
| Community health worker |  | 49 (40–57) | 45 (36 – 55) | 1.0 (0.5-2.2) | 0.9 |  |  |
| **Mode of transport to health facility** | 1,238 |  |  |  |  |  |  |
| On foot |  | 53 (50–56) | 52 (47 – 58) | 1 |  |  |  |
| Bicycle |  | 58 (45–72) | 53 (31 – 74) | 1.0 (0.4-2.5) | 0.9 |  |  |
| Public transport/motorcycle |  | 51 (44–58) | 44 (25 – 63) | 0.7 (0.4-1.4) | 0.3 |  |  |
| **Heard malaria message** | 1,237 |  |  |  |  |  |  |
| No |  | 49 (45–53) | 48 (40 – 55) | 1 |  | 1 |  |
| Yes |  | 56 (52–60) | 54 (46 – 62) | 1.3 (0.9-1.7) | 0.1 | 1.1(0.8-1.6) | 0.5 |
| **Perception of malaria** | 1,236 |  |  |  |  |  |  |
| Not a serious health problem |  | 47 (33–61) | 43 (27 – 60) | 1 |  |  |  |
| Somewhat serious |  | 45 (38–53) | 47 (38 – 56) | 1.2 (0.5-2.9) | 0.7 |  |  |
| Very serious |  | 55 (52–58) | 52 (44 – 60) | 1.4 (0.6-3.3) | 0.4 |  |  |
| **Mention fever symptom** | 1,242 |  |  |  |  |  |  |
| No |  | 53 (46–60) | 49 (36 – 61) | 1 |  |  |  |
| Yes |  | 53 (50–56) | 51 (44 – 59) | 1.1 (0.6-1.9) | 0.7 |  |  |
| **Is malaria treatable** | 1,225 |  |  |  |  |  |  |
| No |  | 41 (34–47) | 36 (29 – 44) | 1 |  |  |  |
| Yes |  | 55 (52–58) | 54 (46 – 61) | 2.0 (1.4-2.9) | **0.001** | 2.2 (1.4-3.5) | **0.004** |
| **Biological child** | 1,230 |  |  |  |  |  |  |
| No |  | 53 (41–66) | 44 (24 – 65) | 1 |  |  |  |
| Yes |  | 53 (50–56) | 51 (44 – 59) | 1.3 (0.5-3.3) | 0.5 |  |  |
| ^1^%; Median (Q1, Q3) | | | |  |  |  |  |
| Abbreviation: CI = Confidence Interval | | | |  |  |  |  |

mRDT: Malaria Rapid Diagnostic Test
