# Supplementary material for: Prevalence of antimalarial drug resistance markers and factors associated with Plasmodium falciparum infection in asymptomatic children prior to rectal artesunate implementation in Kapolowe Health District, Democratic Republic of the Congo
Source: Malar J. 2026 Jun 29;25:332. doi: 10.1186/s12936-026-06016-6 (PMC13584420; doi:10.1186/s12936-026-06016-6)
Supplement: Supplementary file 2 [file 12936_2026_6016_MOESM2_ESM.docx]

**Table S2. Frequencies of all non-synonymous mutations detected in sequenced samples of Pfkelch13 gene**

| **Mutation** | **Amino acid change** | **n** | **% (n/395)** | **95% CI** |
| --- | --- | --- | --- | --- |
| K189T | Lys → Thr | 78 | 19.8 | 16.1–23.1 |
| K189N | Lys → Asn | 12 | 3.0 | 1.8–5.2 |
| L258M | Leu → Met | 10 | 2.5 | 1.4–4.6 |
| H136N | His → Asn | 8 | 2.0 | 1.0–3.1 |
| R255K | Arg → Lys | 8 | 2.0 | 1.0–3.1 |
| A578S | Ala → Ser | 7 | 1.8 | 0.9–3.6 |
| S36T | Ser → Thr | 1 | 0.3 | 0.0–1.4 |
| S50R | Ser → Arg | 1 | 0.3 | 0.0–1.4 |
| V83I | Val → Ile | 2 | 0.5 | 0.1–1.8 |
| D93G | Asp → Gly | 1 | 0.3 | 0.0–1.4 |
| L99V | Leu → Val | 1 | 0.3 | 0.0–1.4 |
| G112E | Gly → Glu | 1 | 0.3 | 0.0–1.4 |
| K114Q | Lys → Gln | 1 | 0.3 | 0.0–1.4 |
| K124N | Lys → Asn | 2 | 0.5 | 0.1–1.8 |
| A174S | Ala → Ser | 1 | 0.3 | 0.0–1.4 |
| K260Q | Lys → Gln | 1 | 0.3 | 0.0–1.4 |
| N264T | Asn → Thr | 1 | 0.3 | 0.0–1.4 |
| I291T | Ile → Thr | 1 | 0.3 | 0.0–1.4 |
| E297G | Glu → Gly | 1 | 0.3 | 0.0–1.4 |
| K324E | Lys → Glu | 1 | 0.3 | 0.0–1.4 |
| I352V | Ile → Val | 1 | 0.3 | 0.0–1.4 |
| I354V | Ile → Val | 1 | 0.3 | 0.0–1.4 |
| G358E | Gly → Glu | 1 | 0.3 | 0.0–1.4 |
| I360T | Ile → Thr | 1 | 0.3 | 0.0–1.4 |
| I376M | Ile → Met | 1 | 0.3 | 0.0–1.4 |
| E377K | Glu → Lys | 1 | 0.3 | 0.0–1.4 |
| E377G | Glu → Gly | 1 | 0.3 | 0.0–1.4 |
| K430E | Lys → Glu | 1 | 0.3 | 0.0–1.4 |
| C473R | Cys → Arg | 1 | 0.3 | 0.0–1.4 |
| T478S | Thr → Ser | 1 | 0.3 | 0.0–1.4 |
| V494A | Val → Ala | 1 | 0.3 | 0.0–1.4 |
| G533D | Gly → Asp | 1 | 0.3 | 0.0–1.4 |
| V534A | Val → Ala | 1 | 0.3 | 0.0–1.4 |
| D547N | Asp → Asn | 1 | 0.3 | 0.0–1.4 |
| S577L | Ser → Leu | 1 | 0.3 | 0.0–1.4 |
| V603A | Val → Ala | 1 | 0.3 | 0.0–1.4 |
| Q613E | Gln → Glu | 1 | 0.3 | 0.0–1.4 |
| F656S | Phe → Ser | 1 | 0.3 | 0.0–1.4 |
| G674E | Gly → Glu | 1 | 0.3 | 0.0–1.4 |
| G690S | Gly → Ser | 1 | 0.3 | 0.0–1.4 |
| F698S | Phe → Ser | 1 | 0.3 | 0.0–1.4 |
| F699L | Phe → Leu | 1 | 0.3 | 0.0–1.4 |
